# Using organoids to investigate human endometrial receptivity

**DOI:** 10.3389/fendo.2023.1158515

**Published:** 2023-08-24

**Authors:** Junhan Guo, Wei Zhou, Michaela Sacco, Poppy Downing, Evdokia Dimitriadis, Feifei Zhao

**Affiliations:** ^1^ Center for Reproductive Medicine, Henan Key Laboratory of Reproduction and Genetics, The First Affiliated Hospital of Zhengzhou University, Zhengzhou, China; ^2^ Department of Obstetrics and Gynaecology, University of Melbourne, Parkville, VIC, Australia; ^3^ Gynaecology Research Centre, Royal Women’s Hospital, Parkville, VIC, Australia

**Keywords:** organoids, endometrial receptivity, implantation, organoid secretion, endometrial epithelial cell

## Abstract

The human endometrium is only receptive to an implanting blastocyst in the mid-secretory phase of each menstrual cycle. Such time-dependent alterations in function require intricate interplay of various factors, largely coordinated by estrogen and progesterone. Abnormal endometrial receptivity is thought to contribute to two-thirds of the implantation failure in humans and therefore significantly hindering IVF success. Despite the incontrovertible importance of endometrial receptivity in implantation, the precise mechanisms involved in the regulation of endometrial receptivity remain poorly defined. This is mainly due to a lack of proper *in vitro* models that recapitulate the *in vivo* environment of the receptive human endometrium. Organoids were recently established from human endometrium with promising features to better mimic the receptive phase. Endometrial organoids show long-term expandability and the capability to preserve the structural and functional characteristics of the endometrial tissue of origin. This three-dimensional model maintains a good responsiveness to steroid hormones *in vitro* and replicates key morphological features of the receptive endometrium *in vivo*, including pinopodes and pseudostratified epithelium. Here, we review the current findings of endometrial organoid studies that have been focused on investigating endometrial receptivity and place an emphasis on methods to further refine and improve this model.

## Introduction

Endometrial receptivity refers to the functional and morphological change of endometrium in the mid-secretory phase to promote a healthy blastocyst to attach, invade, and develop (1). The transition of endometrial receptivity is mainly coordinated by estrogen and progesterone (2). While estrogen is essential to support the regeneration of endometrium after menstruation, progesterone attenuates the proliferative effects of estrogen and primes endometrial cells into a receptive status (3). In particular, the endometrial epithelium which comprises luminal and glandular epithelium undergoes unique functional transitions during receptivity. While the luminal epithelium remodels the surface molecules to become adhesive to the blastocyst, the glandular epithelium enhances secretory activities and releases essential factors apically into the endometrial cavity that can act on both the blastocyst and luminal epithelium to improve blastocyst adhesion (4). As a menstruating species, human endometrium possesses unique patterns of regulation for receptivity that are not typically found in animal models, limiting the applicability of animal models to study human reproduction (5–7). Due to a lack of adequate models, our understanding of the mechanisms that regulate endometrial receptivity in humans remain poorly defined. For instance, it is not fully understood how glands and their basal and apical secretions influence blastocyst adhesion, stromal cell decidualization, and subsequent blastocyst invasion. In addition, how luminal epithelium loses its barrier function and interacts with an implanting blastocyst to facilitate the initial adhesion process remains uncertain.

Recently organoids have been developed from human endometrium. They hold considerable potential to explore endometrial functions. Endometrial organoids are derived from epithelial cells. Endometrial epithelial fragments that consist of glandular and luminal cells, self-organize to form three-dimensional gland-like structures within Matrigel scaffolds. The culture system established is based on a generic expansion medium that assists in the development of other tissue organoids in humans (8). This includes factors that activate the Wnt pathway (R-Spondin-1) and inhibit the transforming growth factor β (TGF-β) (Nicotinamide and A83-01) and bone morphogenetic protein pathways (Noggin) (9, 10). Significant improvement in organoid yield is recorded by adding stromal cell secreted factors FGF10 and HGF that are commonly present in the human endometrium (8). Under this optimized culture system, organoids show long-term expandability and maintain responsiveness to hormones that mimic changes in the mid-secretory phase endometrium (11).

Organoids have been established from human endometrium with pathologies known to affect endometrial receptivity (12, 13). This includes endometrial cancer (14), endometriosis (14), adenomyosis (15, 16), and primary infertility (17). Characterization of these patient-derived endometrial organoids have revealed remarkable similarities compared to their tissues of origin (14). In this context, endometrial cancer derived organoids carry the same genetic mutations, even after long-term culture (14). Organoids established from ectopic endometrium exhibit invasive phenotypes, a pattern seen in ectopic pregnancy *in vivo* (14). Furthermore, organoids established from primary infertile endometrium show an abnormal response to progesterone and cyclic adenosine monophosphate (cAMP) compared to fertile organoids *in vitro*, suggesting a dysregulation in the functional transition towards receptivity (17). All these characteristics of endometrial organoids provide an accessible way to determine the mechanisms for abnormalities in receptivity transition seen in the endometrium in patients. We review current endometrial organoid studies that mimic the mid-secretory phase endometrium and accordingly summarize the challenges and improvements required in this area.

## Using organoids to study endometrial epithelial cell secretions

It is well characterized that endometrial organoids exhibit similar apicobasal polarity compared to endometrial glands (8, 14, 18). Accordingly, immunostaining of basal membrane markers Laminin and Collagen type IV alpha 1 chain (COL4A1) reveals a similar basolateral localization in both endometrial organoids and endometrial glands (8, 17, 19). Electron microscopy further confirms that apical structures of endometrial glands, such as microvilli and cilia (20), similarly face the inner center of the organoids (21). Such polarity of the endometrial organoids allows the separate collection of apical and basal secretions for further investigation.

Organoid apical secretions are representative of *in vivo* endometrial epithelial cell secretions that contribute to the makeup of uterine fluid that bathes the endometrial environment. Recently, a high-throughput centrifugation approach for the large-scale collection of intraorganoid fluid (IOF) representing organoid apical secretions was developed (22). In brief, organoids are gently vortexed and centrifuged to release the IOF captured inside organoids without compromising organoid cell integrity (22). This approach was compared with micromanipulation for IOF collection and demonstrated similar metabolomic profiles between the two IOF collection procedures (22). Using this high-throughput centrifugation approach, a recent study has compared the apical protein secretion profiles of fertile and primary infertile organoids treated with hormones to model the receptive phase (17). This study identified a total of 1150 proteins in both fertile and primary infertile IOF, of which 150 were significantly changed when using a threshold of 1.5-fold change (17). Among these 150 proteins, 131 are decreased in primary infertile IOF, indicating an overall dysregulation of apical protein secretions in the infertile group (17). The physiological relevance of these proteins in the IOF is promising, with 82% of these 131 proteins previously identified in healthy secretory phase uterine fluid (17, 23). Notably, proteins associated with human endometrial receptivity include Dipeptidyl peptidase 4 (DPP4), a membrane-bound glycoprotein that is highly expressed in the mid-secretory phase endometrium and influences glandular differentiation (24, 25). DPP4 is included as a reliable receptivity marker in a few studies, including the endometrial receptivity array (25, 26). Another dysregulated protein is Heat Shock Protein Family A (Hsp70) Member 9 (HSPA9). HSPA9 is localized to the endometrial epithelial cell surface and functionally it facilitates endometrial epithelial cell adhesiveness to trophoblast cells (27). Further investigations of these organoid apical secreted proteins will likely uncover new biomarkers and treatment targets for endometrial receptivity that are epithelial cell specific. Of note, uterine fluid collected either via uterine lavage or aspiration can be challenging for precise quantification of endometrial secreted proteins due to saline and/or blood contaminations, presenting limitations of the applicability of data derived from these solutions. Comparatively, for IOF collected from organoids, the volume can be controlled. Organoids also show long-term expandability which allows for large-scale collection of IOF if required (8).

By contrast, organoid basal secretions are representative of *in vivo* endometrial epithelial cell basal secretions that interact with surrounding stromal cells. Organoid basal secretions are released into the culture medium and can be collected and analyzed. A recent study has characterized microRNAs in extracellular vesicles secreted basally by organoids developed from eutopic endometrium of women with adenomyosis (15). Accordingly, organoids are treated with hormones to facilitate secretory-phase differentiation before the collection of culture media to harvest extracellular vesicles for microRNA sequencing (15). A total of 80 microRNAs have been identified (15), including miR-10a-5p, miR-92a-3p and miR-92b-3p, which are known to represent the mid-secretory phase endometrium (28). The mechanisms underpinning the uptake of extracellular vesicles by surrounding endometrial cells remain to be addressed. In another recent study, the basal secretions of endometrial organoid proteins was determined by Mass spectrometry (29). This studied identified 124 proteins; 90 of which have altered abundance upon progesterone and prostaglandin E2 treatment compared to vehicle control (29). Selective analysis of these changed proteins have identified cystatin C as an important regulator for decidualization using primary endometrial stromal cells (29). Therefore, endometrial organoids serve as a desirable model to investigate gland-stromal communication.

## Using organoid-derived epithelial monolayers to mimic endometrial luminal epithelial cells during receptivity

Endometrial luminal epithelial cells are believed to be derived from PDGFRb-positive stromal cells undergoing mesenchymal to epithelial transition during endometrial repair (30). During the mid-secretory phase, these surface-located epithelial cells undergo morphological and molecular changes, termed “plasma membrane transformation”, to become receptive to blastocyst attachment (31). Dysregulation of these changes in the membrane has been recognized as one of the key contributors to implantation failure (4). As an *in vitro* approach to investigate luminal epithelial cell adhesiveness, Ishikawa cells have been widely used as a receptive endometrial epithelial cell line. However, being derived from endometrial adenocarcinoma, Ishikawa cells also maintain some features of cancer cells and their cellular makeup is likely different compared to normal luminal epithelium (31, 32). Alternatively, primary endometrial epithelial cells are isolated from endometrial biopsies and seeded onto culture plates to generate epithelial cell monolayers (33). These epithelial cell monolayers can then be transfected with siRNAs or plasmids for target-specific investigation. One obvious technical limitation of this endometrial model is that primary endometrial epithelial cells cannot be passaged and stored. Therefore, this approach relies on endometrial biopsy collection.

Endometrial organoids can be used similarly to primary endometrial epithelial cells to generate epithelial cell monolayers in a sustainable manner. In this context, it has been shown that primary endometrial epithelial cell monolayers and organoid derived epithelial cell monolayers respond similarly to miR-29c overexpression when culturing under the same medium consisting of 10% (v/v) fetal calf serum (17, 34). In another study, organoid derived epithelial cell monolayers are treated with hormones and a Wnt pathway inhibitor XAV939 to facilitate the functional transition towards the mid-secretory phase (35). The Wnt pathway inhibitor XAV939 is used to treat monolayers since most Wnt genes are downregulated in the secretory phase of human endometrium compared to the proliferative phase (36). Compared to hormone treatment alone, adding XAV939 further increases the expression of receptivity genes including Leukemia Inhibitory Factor, DPP4 and Glutathione peroxidase 3 (35). Treated organoid monolayers also express ciliated epithelial cell marker acetylated α-tubulin (35). Functionally, compared to untreated endometrial organoid monolayers, treated organoid monolayers show improved adhesiveness to blastoids which are a human peri-implantation blastocyst model (35). Moreover, the contraceptive agent levonorgestrel significantly reduced the adhesiveness of treated organoid monolayers to blastoids suggesting it was likely mediated by progesterone (35, 37).

It is worth noting that although organoid derived monolayers serve as a valuable model to mimic endometrial luminal epithelium, they are substantially different. Organoid derived monolayers represent a mixed epithelial cell population consisting of both luminal and glandular epithelial cells (18, 38). A substantial subpopulation of organoid monolayer cells still express the glandular epithelial marker Forkhead box A2 (35). Introducing decidualized stromal cell secreted factors, such as prolactin, may improve the formation of luminal epithelial cells (8) since they actively interact with stromal cells beneath the luminal surface *in vivo*. Adding another layer of complexity, although organoid cells seeded on Matrigel pre-coated wells show similar basal membrane marker localization compared to endometrial luminal epithelium *in vivo* (17), the apicobasal polarity of organoid derived monolayers remain to be fully characterized. Further investigations into the expression of receptivity markers upon hormone and blastocyst secreted factor treatments are also needed to improve this model.

## Assembling an endometrial-like construct *in vitro*, what is the challenge?

It follows that an ideal model to investigate endometrial receptivity *in vitro* should at least include the main endometrial cell types. In this regard, stromal fibroblasts comprise the largest proportion of cells in the endometrium and actively interact with luminal and glandular epithelial cells in preparation for implantation in the mid-secretory phase (39). Gene Ontology analysis has identified enriched gene sets for stromal cell interactions in initial glandular digests that are missing in cultured endometrial organoids (8). Stromal cells can be collected during the enzymatic digestion of endometrial biopsies for the establishment of organoids, thus making it possible to assemble matched stromal cells and organoids from the same donor (8). Recent work has trialed this in an assembloid model, in which organoids and stromal cells from mid-secretory phase endometrial biopsies are combined in hydrogel to form an endometrial-like 3D construct (40). Assembloids are cultured in organoid expansion medium and treated with estrogen, Medroxyprogesterone acetate (MPA, a stable progestogen) and cAMP to stimulate decidualization. Following decidualization, a day 5 human blastocyst is introduced to determine blastocyst expansion and invasion into the assembloid (40). This study revealed a divergence of both stromal cells and epithelial cells into differentiated and senescent subpopulations and further elucidated active receptor-ligand interactions between certain subpopulations of stromal cells and organoid epithelial cells (40). In addition, acute senescence in epithelial cells and stromal cells have been shown to lead to the production of distinct secretomes critical for implantation (40). Such cellular complexity of the assembloids represent mid-secretory phase endometrium and have consistently identified the importance of proper decidual senescence on blastocyst implantation (41). However, assessment of long term culturing of assembloids requires further experimentation since endometrial organoid culture medium does not favor stromal cell growth (8). Additionally, replacement of the collagen enriched hydrogel matrix with recently developed human endometrium-derived hydrogel may further improve the physiological relevance of assembloids (42).

What is missing in this co-culture model with human blastocysts is the luminal epithelium – blastocysts need to breach the luminal epithelium to invade into a decidualizing stroma. Therefore, a hormone responsive epithelial cell monolayer should be seeded on top of the assembloid to mimic the luminal epithelial cells, similar to a recently proposed endometrial 3D model (43). As mentioned above, organoid-derived epithelial monolayers can be used to mimic the luminal epithelium. Adding immune cells is another challenge in establishing an endometrial-like construct *in vitro* which is yet to be trialed. Immune cells play a significant role in endometrial receptivity (44). For example, increasing number of natural killer cells have been recorded in human endometrium during the mid-secretory phase (45). These cells are appropriately activated to release essential cytokines, support decidualization, eliminate senescent decidual cells and adapt immune response for the implanting blastocyst (41, 44, 46). However, immune cells are more difficult to culture and may require certain cytokines, including interleukins (47).

## Inconsistent culture systems across studies for organoid differentiation

Although it is well accepted that endometrial organoids maintain responsiveness to hormones, the methodology of hormone treatment varies across studies (**Table 1**). To trigger differentiation, all studies use estrogen and progesterone (or MPA); however, the concentration and duration change across different culture systems (**Table 1**). Overall, the duration of hormone treatment is much shorter compared to a natural menstrual cycle. The longest period of hormone treatment in culture is 14 days and lower concentrations of estrogen and progesterone are used compared to other organoid studies (**Table 1**) (18). Culturing organoids for a long period of time has two main challenges. The first is that organoids growing too large are prone to folding in on themselves and begin to die (11). In addition, the stability of commercialized Matrigel (356231, Corning) has only been tested for a maximum period of 14 days at 37°C. Therefore, culturing organoids for too long may disintegrate Matrigel structure and deplete essential nutrients. These limitations may be overcome by new support matrices including a recently developed matrix from healthy human endometrial tissues (42, 53). To further promote organoid differentiation, most studies have introduced cAMP to the differentiation medium (**Table 1**). It has been verified that adding cAMP can further stimulate the expression of secretory phase genes compared to estrogen and progesterone treatment alone (8, 38). However, it seems cAMP treatment does not increase the number of secretory cells (38). Overall, while there is a consensus to use the combination of estrogen, progesterone and cAMP for organoid differentiation (**Table 1**), the conditions are not yet standardized.

**Table 1:** Human endometrial organoid differentiation by hormonal stimulation.

Organoid differentiation by hormonal stimulation	Reference	Year
Organoid expansion medium only for 4d, 10nM estrogen for 2d, 10nM estrogen +1µM progesterone+1µM cAMP for 4d	(8)	2017
1nM estrogen for 7d, 0.1nM estrogen+50ng/mL (0.159nM) progesterone for 7d	(18)	2017
Organoid expansion medium only for 4d, 10nM estrogen for 2d, 10nM estrogen+1µM MPA+1µM cAMP for 6d	(38)	2019
10nM estrogen for 4d, 1µM estrogen+1µM MPA+0.5mM cAMP for 4d (co-cultured with stormal cells for decidualization)	(40)	2021
Organoid expansion medium only for 4d, 10nM estrogen for 2d, 10nM estrogen+1µM progesterone+1µM cAMP for 4d	(48)	2021
Organoid expansion medium+Rho kinase inhibitor+CHIR 99021 for 10d, 10nM estrogen for 2d (R-spondin-1 free), 10nM estrogen+1µM progesterone+100µg/mL (304µM) cAMP for 4d (R-spondin-1 free) (introduced NOTCH and WNT inhibitors)	(49)	2021
Organoid expansion medium only for 4d, 10nM estrogen for 2d, 10nM estrogen+1µM MPA for 3d	(50)	2022
Organoid expansion medium only for 2d, 10nM estrogen for 2d, 10nM estrogen+1µM progesterone+250µM cAMP for 4d	(35)	2022
Organoid expansion medium only for 4d, 10nM estrogen for 2d, 10nM estrogen+1µM progesterone+1µM cAMP for 6d	(51)	2022
Organoid expansion medium only for 4d, 10nM estrogen for 2d, 10nM estrogen+1µM progesterone+1µM cAMP for 4d	(15)	2022
Organoid expansion medium only for 4d, 10nM estrogen for 2d, 10nM estrogen+1µM progesterone+1µM cAMP for 4d	(52)	2022
Organoid expansion medium only for 4d, 10nM estrogen for 2d, 10nM estrogen+1µM MPA+1µM cAMP for 4d	(17)	2022

Another obvious difference in hormone stimulus is that while the peak concentration of estrogen in serum reduces from the proliferative phase to the mid-secretory phase in a natural menstrual cycle (54), it is maintained at a stable level in most organoid cultures for differentiation (**Table 1**). This may account for why the number of proliferating cells and ciliated cells, that should be greatly decreased in the mid-secretory phase, are not reduced in organoids following differentiation (38). Only one study has used a lower concentration of estrogen when adding progesterone to endometrial organoids (**Table 1**) (18). Promisingly, proliferating cells are significantly reduced compared to estrogen treatment alone (18).

Aside from hormone treatment, the culture medium for organoid differentiation also warrants further optimization. Organoid expansion medium, which has been used in all organoid differentiation studies (**Table 1**), contains WNT pathway activator R-Spondin-1 and TGF-β inhibitors Nicotinamide and A83-01. It is contradictory to *in vivo* mid-secretory phase physiological conditions in which the WNT pathway is downregulated and the TGF-β pathway is active (36, 55, 56). A recent study has trialed a base medium (advanced DMEM/F12+Primocin+Glutamax) with the addition of only B27 and Insulin-transferrin-selenium to support the growth of organoid cells (29). Briefly, organoids are established under expansion medium and then cultured in base medium with hormones for differentiation (29). No effects on cell growth have been observed and promisingly, the expression of hormone responsive genes are changed as expected, including ectonucleotide pyrophosphatase/phosphodiesterase family member 3 and progesterone receptor (PGR) (29). Currently there is no clear consensus on which culture medium best models the mid-secretory phase endometrium. A more refined treatment of hormones and culture medium are required to closely resemble the *in vivo* changes and a set of receptivity markers should be tested for optimization.

## Stability of endometrial organoids between menstrual cycles

Monitoring endometrial status between menstrual cycles are pivotal for clinical applications. In the IVF clinical setting, frozen embryo transfer is delivered in a different cycle after the failure of fresh embryo transfer (57). Although it has been trialed (58), consenting patients for multiple endometrial biopsy collections is not easy. Excitingly, organoids can be established from menstrual fluid with a success rate of 87% (48). Comparison of organoids developed from scratched endometrial biopsies and paired menstrual fluid from the same menstrual cycle have demonstrated similarities over their transcriptome profiles, as revealed by principal component analysis on top 2000 genes clustering (48). Similar responses to hormones have also been identified by examining the production and secretion of uterine milk proteins (48). Collection of menstrual fluid is completely non-invasive and therefore offers an accessible way to determine endometrial function between menstrual cycles from a large population of women. Adding to this advantage is the fact that organoids are only comprised of epithelial cells (8). This provides a more targeted approach to assess endometrial receptivity after hormone treatment of organoids. The stability of menstrual fluid derived organoid phenotype between menstrual cycles is yet to be determined. For future analysis, we encourage standardization of an epithelial cell specific gene set for organoid differentiation assessment.

## Do endometrial organoids recapitulate the complexity of the endometrial epithelium in the mid-secretory phase *in vivo*?

Although the conditions require further optimization for endometrial organoid differentiation, organoids respond to ovarian hormones in a similar way compared to the endometrial epithelium *in vivo*. In this context, organoids treated with estrogen increase the expression of PGR and addition of progesterone minimizes PGR levels (38). Such changes recapitulate the *in vivo* transition of PGR in both glandular and luminal epithelium from proliferative phase to mid-secretory phase (59, 60). Progesterone and cAMP treatment in organoids also increase the expression of a set of receptive genes including *LIF*, *HSD17B2*, *PAEP*, *GPX3* and *FOXO1* (17, 38), all reproducing *in vivo* changes in the mid-secretory phase human endometrium (4, 18, 38, 61, 62). Among these genes, *PAEP* and *GPX3* are identified as subphase defining markers that show abrupt changes of expression from early-secretory phase to mid-secretory phase (63).

Single cell sequencing has been applied to define different epithelial cell types in human endometrial organoids (38, 49). Six cell types, namely stem, ciliated, unciliated, epithelial, proliferative and secretory epithelial cells are identified in organoids that have been cultured *in vitro* for 12 days under control, E2+MPA or E2+MPA+cAMP treatments (38). Compared to control, both MPA and MPA+cAMP treatments reduce the stem cell population and accordingly trigger cell differentiation similar to mid-secretory phase endometrial epithelium *in vivo* (38). There is also an obvious increase in ciliated cell number driven by estrogen treatment (1.5% in control versus 34.4% in E2 treated organoids) and introduction of MPA reduces this cell population compared to estrogen treatment alone (38). These changes are in accordance with previous findings *in vivo*; the population of ciliated endometrial epithelial cells increase in response to high levels of estrogen, reach maximum in the proliferative phase, and reduce in the secretory phase due to rising levels of progesterone (64, 65).

In another study using single cell sequencing, a comparison of *in vitro* endometrial organoids and *in vivo* endometrial epithelium is made (49). Briefly, two single cell sequencing datasets, *in vivo* endometrial epithelium from healthy endometrium across the menstrual cycle and endometrial organoids treated with different combinations of hormones are established (49). Using a logistic regression model, the organoid single cell sequencing dataset is assigned to the *in vivo* endometrial epithelium dataset to project cell clusters. Upon estrogen and progesterone treatment, organoids undergo differentiation with secretory and ciliated cell populations matching closely (26.5% and 99.65% respectively) with *in vivo* counterparts (49). This study also established a cell–cell communication pipeline based on single cell sequencing and spatial transcriptomics data to uncover key pathways driving epithelial cell differentiation under *in vivo* microenvironment (49). It predicts that WNT and NOTCH pathways are differently regulated in glandular and luminal epithelium to shape cell identity and function, under the regulation of ovarian hormones (49). To test this prediction, human endometrial organoids are established and treated with estrogen, progesterone, prolactin and cAMP. Compared to *in vivo*, similar targets of WNT and NOTCH pathways are activated/inhibited in secretory (glandular) and ciliated (luminal) epithelium in organoids (49). Furthermore, targeting WNT and NOTCH pathways in organoids with their respective inhibitors are able to confirm their functions on ciliated and secretory cell differentiation, which is consistent as predicted *in vivo* (49). This study proved that WNT pathway activation and NOTCH pathway inhibition is essential for luminal epithelial cell differentiation. While for glandular epithelial cell differentiation, the opposite regulation of these two pathways are required (49).

Overall, organoids serve as a great *in vitro* model to study endometrial epithelial cell differentiation. However, we encourage caution in direct extrapolation of organoid response to progesterone to the equivalent events occurring within mid-secretory phase endometrium. It is thought that in endometrial tissue, most of the effects of progesterone on epithelium come via the stromal cells which have much higher levels of PGR in the mid-secretory phase (66).

## Conclusion

Endometrial receptivity is vital for successful blastocyst implantation. In humans, the precise mechanisms involved in the regulation of endometrial receptivity remain to be elucidated. This is largely attributed to the lack of systems to closely resemble mid-secretory phase endometrium. Endometrial organoids have recently been established from women with normal fertility and endometrial diseases that affect receptivity. Epithelial cells are a key driver of endometrial disease. Endometrial organoids consist only of epithelial cells and importantly retain their key features from tissue of origin, even after a long period of culture, thus making them an ideal model to study mechanisms driving endometrial diseases and their relations to receptivity *in vitro*. Endometrial organoids also offer a sustainable approach to assemble endometrial-like 3D constructs to study the impact of the embryo or its signals on endometrial responses. We have proposed several challenges to improve organoid model. Resolving these challenges promises to advance our understanding of the mechanisms that exert regulatory control over the functional transition of the human endometrium during receptivity.

## Author contributions

All authors made substantial contributions to the conception of this review. JG, WZ, and FZ generated the initial draft of the manuscript, and it was critically revised by MS, PD, ED and FZ. All authors approved the final version and submission of this review.
